# Emphysematous Cystitis

**DOI:** 10.5334/jbsr.1192

**Published:** 2018-10-12

**Authors:** Sven Dekeyzer, Brecht Houthoofd

**Affiliations:** 1 Universitair Ziekenhuis Antwerpen (UZA), BE; 2 Sint-Andriesziekenhuis, Tielt, BE

**Keywords:** emhysematous cystitis, bladder, urinary tract, infection

**Figure 1:**
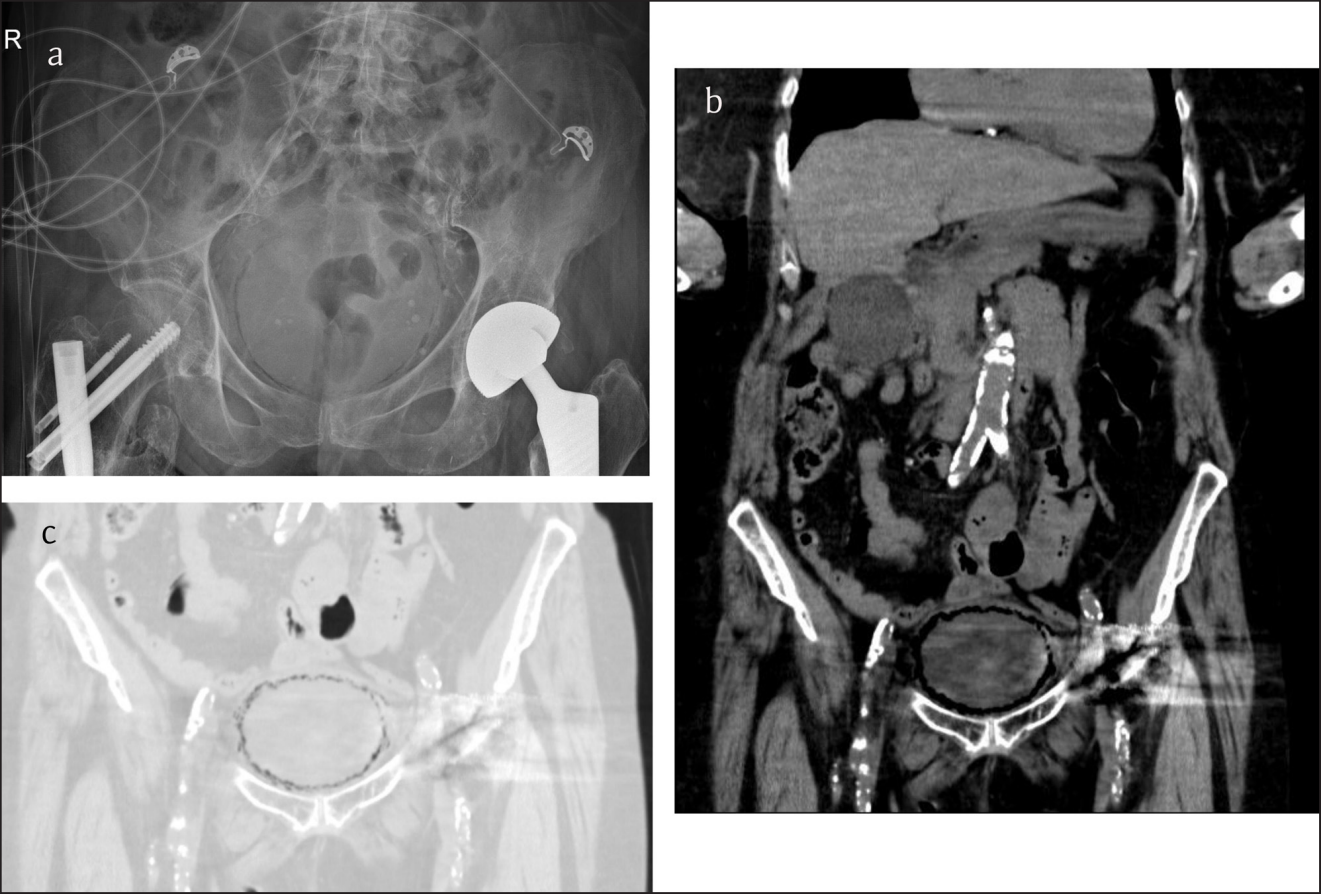
Conventional radiograph **(a)** in a case of emphysematous cystitis shows sharply demarcated, curvilinear areas of increased lucency in the pelvis. Computed tomography **(b, c)** images more clearly reflect the cobblestone aspect of intramural gas delineating the bladder wall, often likened to a “beaded necklace” appearance.

A 71-year-old woman was brought to the emergency room because of confusion and coffee ground vomitus. Physical examination was unremarkable. The abdomen was soft and non-tender. Laboratory tests showed normochromic normocytic anemia, elevated inflammatory parameters and elevated kidney function tests. Urine analysis showed numerous red blood cells, white blood cells and bacteriae. Abdominal X-ray showed a curvilinear area of radiolucency delineating the bladder wall (Figure 1a). Non-contrast enhanced computed tomography (CT) of the abdomen revealed multiple gas pockets within the bladder wall (Figure 1b and 1c). A diagnosis of emphysematous cystitis was made and the patient was started empirically on intravenous piperacillin/tazobactom. Urine culture grew E. Coli and treatment was switched to intravenous levofloxacin. There was gradual clinical improvement of the patient. Control ultrasonography performed the week after admission showed a normal bladder wall.

Emphysematous cystitis is an uncommon but severe lower urinary tract infection characterized by the accumulation of gas in and around the bladder wall produced by bacterial or fungal fermentation. The most common causative organisms are Escherichia Coli, Enterobacter Aerogenes and Klebsiella Pneumonia. The majority of patients are elderly, female and have type 2 diabetes mellitus. The clinical presentation of emphysematous cystitis is nonspecific and varied, ranging from asymptomatic urinary tract infection to severe peritonitis or septic shock. Emphysematous cystitis requires aggressive treatment with parenteral antibiotics, bladder drainage and control of sugar level and the overall average mortality rate is approximately 7% [1].

Because of the nonspecific presentation, imaging plays an important role in the diagnosis. Plain abdominal X-rays can show a rim of gas lucency outlining the bladder wall and/or air fluid levels within the bladder. Computed tomography (CT) can more accurately define the extent and severity of the disease, can detect cases that are not apparent on plain radiography and can help in differentiating emphysematous cystitis from colovesical fistula, intra-abdominal abcesses, neoplastic disease or emphysematous pyelonephritis.
